# Anti-citrullinated peptide/protein antibody (ACPA)-negative RA shares a large proportion of susceptibility loci with ACPA-positive RA: a meta-analysis of genome-wide association study in a Japanese population

**DOI:** 10.1186/s13075-015-0623-4

**Published:** 2015-04-18

**Authors:** Chikashi Terao, Koichiro Ohmura, Yuta Kochi, Katsunori Ikari, Yukinori Okada, Masakazu Shimizu, Naoshi Nishina, Akari Suzuki, Keiko Myouzen, Takahisa Kawaguchi, Meiko Takahashi, Kiyoshi Takasugi, Akira Murasawa, Shinichi Mizuki, Mitsuhiro Iwahashi, Keiko Funahashi, Masamitsu Natsumeda, Moritoshi Furu, Motomu Hashimoto, Hiromu Ito, Takao Fujii, Kazuhiko Ezawa, Tsukasa Matsubara, Tsutomu Takeuchi, Michiaki Kubo, Ryo Yamada, Atsuo Taniguchi, Hisashi Yamanaka, Shigeki Momohara, Kazuhiko Yamamoto, Tsuneyo Mimori, Fumihiko Matsuda

**Affiliations:** Center for Genomic Medicine, Kyoto University Graduate School of Medicine, Kyoto, Japan; Department of Rheumatology and Clinical Immunology, Kyoto University Graduate School of Medicine, Kyoto, 606-8507 Japan; Laboratory for Autoimmune Diseases, Center for Genomic Medicine, RIKEN, Yokohama, Japan; Institute of Rheumatology, Tokyo Women’s Medical University, Tokyo, Japan; Department of Human Genetics and Disease Diversity, Graduate School of Medical and Dental Sciences, Tokyo Medical and Dental University, Tokyo, Japan; Division of Rheumatology, Department of Internal Medicine, Keio University School of Medicine, Tokyo, Japan; Dohgo Spa Hospital, Matsuyama, Japan; Department of Rheumatology, Niigata Rheumatic Center, Niigata, Japan; The Centre for Rheumatic Diseases, Matsuyama Red Cross Hospital, Matsuyama, Japan; Higashihiroshima Memorial Hospital, Hiroshima, Japan; Pharm C, Matsubara Mayflower Hospital, 944-25 Fujita, Kato City, Hyogo Japan; Kurashiki Sweet Hospital, Kurashiki, Japan; Department of the Control for Rheumatic Diseases, Kyoto University Graduate School of Medicine, Kyoto, Japan; Matsubara Mayflower Hospital, 944-25 Fujita, Kato City, Hyogo Japan; Institut National de la Sante et de la Recherche Medicale (INSERM) Unite U852, Kyoto University Graduate School of Medicine, Kyoto, Japan

## Abstract

**Introduction:**

Although susceptibility genes for anti-citrullinated peptide/protein antibodies (ACPA)-positive rheumatoid arthritis (RA) have been successfully discovered by genome-wide association studies (GWAS), little is known about the genetic background of ACPA-negative RA. We intended to elucidate genetic background of ACPA-negative RA.

**Method:**

We performed a meta-analysis of GWAS comprising 670 ACPA-negative RA and 16,891 controls for 1,948,138 markers, followed by a replication study of the top 35 single nucleotide polymorphisms (SNPs) using 916 cases and 3,764 controls. Inverse-variance method was applied to assess overall effects. To assess overlap of susceptibility loci between ACPA-positive and -negative RA, odds ratios (ORs) of the 21 susceptibility markers to RA in Japanese were compared between the two subsets. In addition, SNPs were stratified by the p-values in GWAS meta-analysis for either ACPA-positive RA or ACPA-negative RA to address the question whether weakly-associated genes were also shared. The correlations between ACPA-positive RA and the subpopulations of ACPA-negative RA (rheumatoid factor (RF)-positive and RF-negative subsets) were also addressed.

**Results:**

Rs6904716 in *LEMD2* of the human leukocyte antigen (HLA) locus showed a borderline association with ACPA-negative RA (overall p = 5.7 × 10^−8^), followed by rs6986423 in *CSMD1* (p = 2.4 × 10^−6^) and rs17727339 in *FCRL3* (p = 1.4 × 10^−5^). ACPA-negative RA showed significant correlations of ORs with ACPA-positive RA for the 21 susceptibility SNPs and non-HLA SNPs with p-values far from significance. These significant correlations with ACPA-positive RA were true for ACPA-negative RF-positive and ACPA-negative RF-negative RA. On the contrary, positive correlations were not observed between the ACPA-negative two subpopulations.

**Conclusion:**

Many of the susceptibility loci were shared between ACPA-positive and -negative RA.

**Electronic supplementary material:**

The online version of this article (doi:10.1186/s13075-015-0623-4) contains supplementary material, which is available to authorized users.

## Introduction

Rheumatoid arthritis (RA) is the most common autoimmune arthritis worldwide with prevalence of around 1%. Genetic factors as well as environmental factors are involved in the development of RA [1]. *HLA*, especially *HLA-DRB1*, is the strongest susceptibility locus to RA beyond ethnicity. Anti-citrullinated peptide/protein antibody (ACPA) is a highly specific autoantibody to RA, which recognizes a broad range of citrullinated peptides and appears in approximately 80% of patients with RA [2-4]. Previous studies addressing the genetic difference between RA subsets with or without ACPA have shown that ACPA-negative RA has different susceptibility human leukocyte antigen (HLA) alleles from ACPA-positive RA [5-8]. A recent study in Europeans also confirmed the different susceptibility HLA alleles in the context of susceptibility effects of amino acid residues [9].

Recent genome-wide association studies (GWAS) have identified more than 40 non-HLA susceptibility loci in Europeans and Asians [10-22] and these studies have also shown overlapped susceptibility loci among different populations [14,22] as well as ethnicity-specific susceptibility loci [12,13,23]. Previous GWAS analyzed ACPA-positive RA or RA regardless of ACPA positivity. Evidence on the genetic background of ACPA-negative RA is quite limited, and there are only a few GWAS addressing ACPA-negative RA from the European population [24,25]. The GWAS failed to identify specific susceptibility loci to ACPA-negative RA beyond the GWAS significance level. Several studies from mainly European countries using candidate gene approach have reported susceptibility markers to ACPA-negative RA [26-30], but none of them were specific to ACPA-negative RA with strong *P*-values, due to their limited numbers of subjects.

In addition to previous reports of different HLA susceptibility alleles to ACPA-positive and -negative RA, one UK study reported different associations of RA susceptibility loci between ACPA-positive and -negative RA [27]. On the contrary, another study from the US reported that ACPA-negative RA shares large fractions of susceptibility loci with ACPA-positive RA except for HLA [31]. Thus, similarities and differences in genetic components between ACPA-negative and -positive RA are inconclusive.

Furthermore, previous studies suggest that ACPA-negative RA has two distinct subsets based on rheumatoid factor (RF) positivity in association with HLA alleles [32,33]. No studies have ever addressed whether the two ACPA-negative subsets share non-HLA susceptibility alleles with ACPA-positive RA or with each other. Moreover, there are no GWAS or candidate gene studies addressing non-HLA locus in ACPA-negative RA from an Asian population, except for a subanalysis of GWAS against RA in a Chinese population [34]. A GWAS of ACPA-negative RA subjects from an Asian population may identify novel susceptibility loci to ACPA-negative RA, which were not found in the previous European GWAS due to lack of power, or which are specific to the Asian population. Asian GWAS would also elucidate overlapping and dissociations of susceptibility loci between ACPA-positive and -negative RA in the Asian population, including detailed analysis for the two subsets of ACPA-negative RA.

Here, to elucidate the genetic background of ACPA-negative RA for the first time in the Asian population, we performed a meta-analysis of GWAS comprising 670 patients with ACPA-negative RA and 16,891 controls in a Japanese population, followed by a replication study of 916 cases and 3,764 controls. We also analyzed genetic correlations between ACPA-positive and -negative RA or the two subsets of ACPA-negative RA based on RF positivity.

## Methods

### Ethics statement

This study was designed in accordance with the Helsinki Declaration. This study was approved by the local ethics committees, namely, Kyoto University Graduate School and Faculty of Medicine, Ethics Committee and Ethics Committees of RIKEN, Tokyo Women’s Medical University Matsuyama Red Cross Hospital, Keio University School of Medicine, Dohgo Spa Hospital, Niigata Rheumatic Center, Higashihiroshima Memorial Hospital, Kurashiki Sweet Hospital and Pharm C, Matsubara Mayflower Hospital. Written informed consent was obtained from all study participants. All data were de-identified and analyzed anonymously.

### Study subjects

A total of 670 patients with ACPA-negative RA and 16,891 controls were enrolled in the three GWAS from RIKEN, Kyoto University, and Tokyo Women’s Medical University, respectively, and 916 cases and 3,764 controls in the replication study. The subjects in GWAS were included in the meta-analysis of RA recently reported from GARNET consortium [14] (RA meta-analysis hereafter). A summary of the participants is presented in Table 1. All of the patients fulfilled American College of Rheumatology (ACR) revised criteria for RA in 1987 [35] or ACR and European League Against Rheumatism (EULAR) classification criteria for RA in 2010 [36].

**Table 1:** **Summary of study subjects**

	**Cases**	**Controls**
**GWAS meta-analysis**		
Number of subjects	670	16,891
Cohort	Kyoto, BBJP, IORRA	BBJP
Age*	60.4 ± 18.9	60.9 ± 12.5
Female ratio	81.6%	44.9%
Genotyping platform	Illumina HumanHap610-Quad BeadChip	Illumina HumanHap610-Quad BeadChip
	Illumina HumanHap300 BeadChip	
	Illumina Human CNV370-Duo BeadChip	
	Affymetrix Genome-wide Human SNP Array 6.0	
RF positivity	46.3%	-
**Replication study**		
Number of subjects	916	3,764
Cohort	Kyoto, BBJP, IORRA	Kyoto University
Age*	62.9 ± 13.7	59.3 ± 14.2
Female ratio	77.8%	48.4
Genotyping platform	Taqman Assay	Illumina HumanHap610-Quad BeadChip
		Illumina HumanHap550 BeadChip
RF positivity**	40.0%	-

*Mean ± standard deviation. **Rheumatoid factor (RF) positivity rate of samples with RF data available. GWAS, genome-wide association studies; BBJP, Biobank Japan; IORRA, cohort of Tokyo Women’s Medical University; SNP, single nucleotide polymorphism.

### ACPA detection

The MESACUP CCP ELISA kit (Medical and Biological Laboratories Co., Ltd, Nagoya, Japan) was used to detect second-generation ACPA in each RA patient, according to the manufacturer’s instructions. A cutoff value of 4.5 U/ml was used to define ACPA positivity.

### RF detection

The serum RF concentrations of samples were quantified using a latex agglutination turbidimetric immunoassay or an ELISA assay. When multiple values for RF had been obtained at different visits, we used the maximum RF value for each patient. The cutoff values of each detection kit in each hospital were employed.

### Genotyping

Microarrays in Illumina Infinium and Affymetrix were used for the meta-analysis of the three GWAS. Detailed information on the arrays was given in the previous report [14]. In the replication study, Taqman assays were performed for genotyping case subjects, and genotype data for controls were extracted from array data of Illumina Infinium HumanHap610-Quad or HumanHap550 (Table 1). Association data for 2,822 patients with ACPA-positive RA and 16,891 controls were obtained from the RA meta-analysis. We applied the same quality-control criteria as the RA meta-analysis including call rate, minor allele frequency, and Hardy-Weinberg disequilibrium: these details were presented in the previous manuscript [14].

### Imputation

Imputed genotype data of ACPA-negative RA patients was extracted from the RA meta-analysis in a Japanese population. Briefly, MACH version 1.0.16 software was used for imputation of genotype data obtained by the GWAS of RA with the Hapmap phase II East Asian panel (JPT and CHB) as reference. As the meta-analysis in the current study included three different GWAS, the imputation was performed separately for data from each GWAS using the same reference panel. A total of 1,948,138 single nucleotide polymorphisms (SNPs) with minor allele frequency >1% and imputation score (Rsq) >0.5 were used for the analysis.

### Thirteen regions associated with ACPA-negative RA in a European population

SNPs in the 13 regions that were reported to be associated with ACPA-negative RA in a European population were extracted from the GWAS meta-analysis. The 13 SNPs that had the strongest associations among the 13 regions were selected as representatives of the regions. We performed a total of 20,000 permutation tests to evaluate empirical *P*-values to obtain the smallest *P*-values <0.01 from 4 of the 13 regions.

### SNP selection for the replication study

SNPs with *P*-values <1 × 10^−4^ in the GWAS meta-analysis and contained in both the Illumina Infinium HumanHap 610-Quad array and the Human Hap 550 array, and for which real-time PCR primers and probes were successfully designed, were selected for the replication study. Rs3889769 was excluded due to difficulty in designing probes for the replication study. The 21 SNPs that were shown to be susceptibility markers in the RA meta-analysis and were contained in both the Illumina Infinium HumanHap 610-Quad array and the Human Hap 550 array were also selected for the replication study to analyze correlation of effect sizes (odds ratio, OR) between ACPA-negative and -positive RA. For *FCRL3*, rs17727339, which had a strong association with ACPA-negative RA, was used in the analysis.

### Assessment of heterogeneity

Heterogeneity among three GWAS or among the GWAS and the replication study was evaluated by the Cochran *Q*-test and *I*^2^.

### Correlation analysis

Effect sizes (ORs) of risk alleles in the 21 SNPs were compared between ACPA-positive and -negative RA by calculating the Spearman correlation coefficient in the GWAS meta-analysis and the replication study. Correlation of effect sizes for non-HLA SNPs in GWAS meta-analysis was analyzed between ACPA-positive and -negative RA, -negative RF-positive or -negative RF-negative RA by Spearman’s correlation coefficient for SNPs pruned by *r*^2^ <0.3 by PLINK with intervals of *P*-values. Data for *r*^2^ was obtained in the Hapmap phase II JPT data. HLA was defined from 25 Mbp to 35 Mbp on chromosome 6 based on NCBI build 36.

### Statistical analysis

Dosage of risk alleles were assessed for associations with susceptibility to ACPA-negative RA by logistic regression analysis. The inverse-variance method was used to combine results of the three GWAS in the meta-analysis assuming a fixed-effect model from study-specific effect sizes (logarithm of ORs) and to combine results of the GWAS meta-analysis and the replication study. The QQ plot was used to assess population structure in the GWAS meta-analysis. Genomic control methods were applied to the test statistics in each of the three GWAS of ACPA-negative RA patients based on the inflation factor calculated in each study. Because the meta-analysis of the three GWAS did not show an inflation factor >1.0, we did not apply genomic control to the results of the meta-analysis. We also performed GWAS meta-analysis using age and sex as covariates. Statistical analyses were performed by R software or PLINK version 1.07 [37]. *P*-values <0.05 and 5 × 10^−8^ were regarded as significant for correlation analyses and GWAS, respectively.

## Results

**Figure 1:**
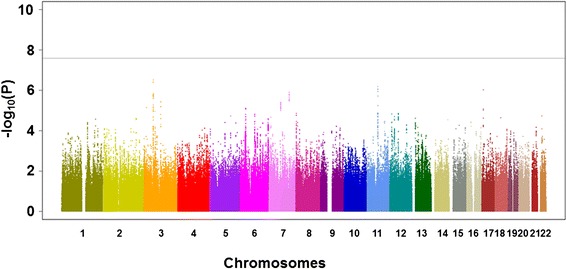
Manhattan plot of meta-analysis of three genome-wide association studies (GWAS) for anti-citrullinated peptide/protein antibody-negative rheumatoid arthritis. The horizontal line indicates the GWAS significance level (*P* = 5 × 10^−8^).

The overall strategies of our study are demonstrated in Additional file 1. First, a total of 670 patients with ACPA-negative RA from three independent cohorts (GARNET Consortium detailed in Methods) and 16,891 controls were genome-scanned with different SNP typing platforms (Table 1) and the data were imputed separately using the same Hapmap phase II East Asian panel as reference and corrected in order to fit meta-analysis as detailed in Methods. The QQ plot of the GWAS meta-analysis did not show evidence of population stratification (lambda = 0.98, Additional file 2). Thus, we did not apply genomic control to our GWAS results. The result of the GWAS meta-analysis is shown in Figure 1 and no markers, including the HLA locus, reached the GWAS significant level. However, when we focused on our results in the GWAS meta-analysis of the genes that had been reported to be associated with ACPA-negative RA in the previous studies, four out of the 13 genes had SNPs with suggestive association (*P* <0.01) (Additional file 3). This ratio was significantly higher than the ratio obtained by chance based on 20,000 permutations (permutation *P* = 0.017). We confirmed that the effect sizes and Manhattan plot in the meta-analysis were almost identical to those in the meta-analysis using age and sex as covariates (Additional files 4 and 5).

Next, we performed a replication study by selecting 35 SNPs that showed suggestive associations with ACPA-negative RA in the GWAS meta-analysis (Additional file 6, *P* <1.0 × 10^−4^; for detail see Methods). An independent set of 916 patients with ACPA-negative RA and 3,764 controls were used for the replication study using the Taqman Assay. As a result, 3 out of the 35 SNPs, namely, rs6904716 in *LEM domain containing 2* (*LEMD2*) on the HLA locus, rs17727339 in *Fc receptor like 3* (*FCRL3*) on chromosome 1, and rs6986423 in *CUB and Sushi multiple domains 1* (*CSMD1*) on chromosome 8, showed the same direction of risk alleles as the GWAS meta-analysis with *P*-values <0.05 (Table 2). None of these three SNPs showed deviation from Hardy-Weinberg equilibrium (*P* ≥0.039). In the combined study using the inverse-variance method, rs6904716, which is contained in the HLA locus, had borderline association (*P* = 5.7 × 10^−8^). These three SNPs did not show significant heterogeneity among the three GWAS and among the GWAS and the replication study (Table 2). While rs6986423 showed a moderate heterogeneity, the *P* -value based on *Q*-statistics was not significant (*P* = 0.076).

**Table 2:** **Association of the top 3 SNPs with ACPA-negative RA among the 35 replicated SNPs**

**SNP**	**Chr**	**Position**	**Gene**	**Exon/intron**	**Ref**	**Var**	**GWAS**				**Replication study**			**Combined study**		
							**Beta**	**SE**	***P***	***I*** ^**2**^ **(%)**	**Beta**	**SE**	***P***	***P***	**OR (95% CI)**	***I*** ^**2**^ **(%)**
rs6904716	6	33849267	*LEMD2*	intron	A	G	−0.309	0.074	2.7 × 10^−5^	0	−0.219	0.061	0.00036	5.7 × 10^−8^	0.77 (0.70, 0.85)	0
rs6986423	8	4621823	*CSMD1*	intron	G	T	−0.248	0.057	1.4 × 10^−5^	55.9	−0.128	0.053	0.017	2.4 × 10^−6^	0.83 (0.77, 0.90)	61.3
rs17727339	1	155946738	*FCRL3*	-	C	T	−0.262	0.066	8.0 × 10^−5^	0	−0.142	0.063	0.025	1.4 × 10^−5^	0.82 (0.75, 0.90)	0

SNP, single nucleotide polymorphism; ACPA, anti-citrullinated peptide/protein antibody; RA, rheumatoid arthritis; GWAS, genome-wide association studies; OR, odds ratio; Chr, chromosome; Ref, reference; Var, variance; SE, standard error.

**Figure 2:**
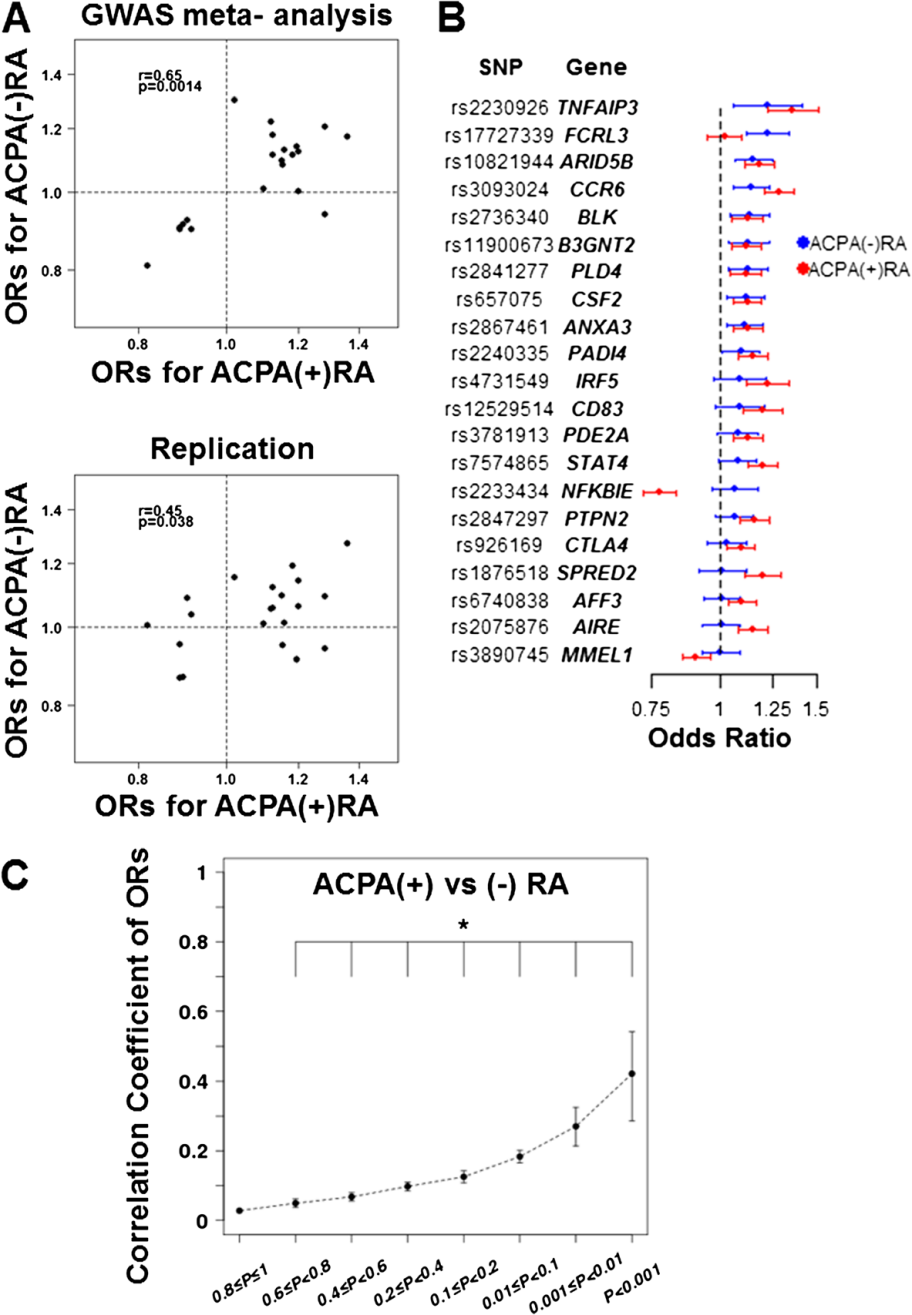
Correlation of odds ratios (ORs) of rheumatoid arthritis (RA) susceptibility single nucleotide polymorphisms (SNPs) for anti-citrullinated peptide/protein antibody (ACPA)-positive RA and those for ACPA-negative RA. **(A)** Correlations of ORs regarding the 21 susceptibility SNPs to RA for ACPA-positive and -negative RA. The upper panel shows the results in the genome-wide association studies (GWAS) meta-analysis and the lower panel shows the results in the replication study. **(B)** ORs of the 21 SNPs in RA-susceptibility loci for ACPA-negative RA in the combined analysis (blue line) and ACPA-positive RA (red line) are plotted. Mean +/− 95% CI are shown. **(C)** Correlation coefficient of ORs between ACPA-positive and -negative RA in terms of SNPs stratified by *P*-values for ACPA-positive RA. *P-values <1.0 × 10^−7^. SNPs in the HLA locus were excluded from this analysis.

Although we did not find any variants associated with ACPA-negative RA with *P*-values beyond the GWAS significance level, we hypothesized that ACPA-negative RA shares a large part of the susceptibility loci with ACPA-positive RA. To test this hypothesis, we compared the ORs of the associated SNPs in ACPA-positive RA with those in ACPA-negative RA. First we selected the 21 non-HLA susceptibility SNPs in the RA meta-analysis in a Japanese population [14] (for detail see Methods). As shown in Figure 2A, ORs of the 21 SNPs for ACPA-negative RA are clearly correlated with those for ACPA-positive RA (*r* = 0.65, *P* = 0.0014, Figure 2A and detailed in Additional file 7). These 21 SNPs were also genotyped for the replication study (916 patients with ACPA-negative RA and 3,764 healthy controls), and again the significant association of ORs between the two RA subsets was obtained (*r* = 0.45, *P* = 0.038, Figure 2A and detailed in Additional file 7). These results strongly suggest that ACPA-negative RA shares non-HLA susceptibility loci with ACPA-positive RA (Figure 2B).

Next, to address the question as to whether weakly associated genes are also shared between ACPA-positive and -negative RA, SNPs were stratified by the *P*-values in the GWAS meta-analysis for either ACPA-positive RA or ACPA-negative RA and correlation coefficients of ORs for the SNPs in each stratified fraction were plotted. As shown in Figure 2C, the smaller the *P*-values, the bigger the correlation coefficients. These results suggest that the susceptibility loci of ACPA-positive RA and ACPA-negative RA are largely overlapped and the effect size of each SNP is similar between the two RA subsets.

**Figure 3:**
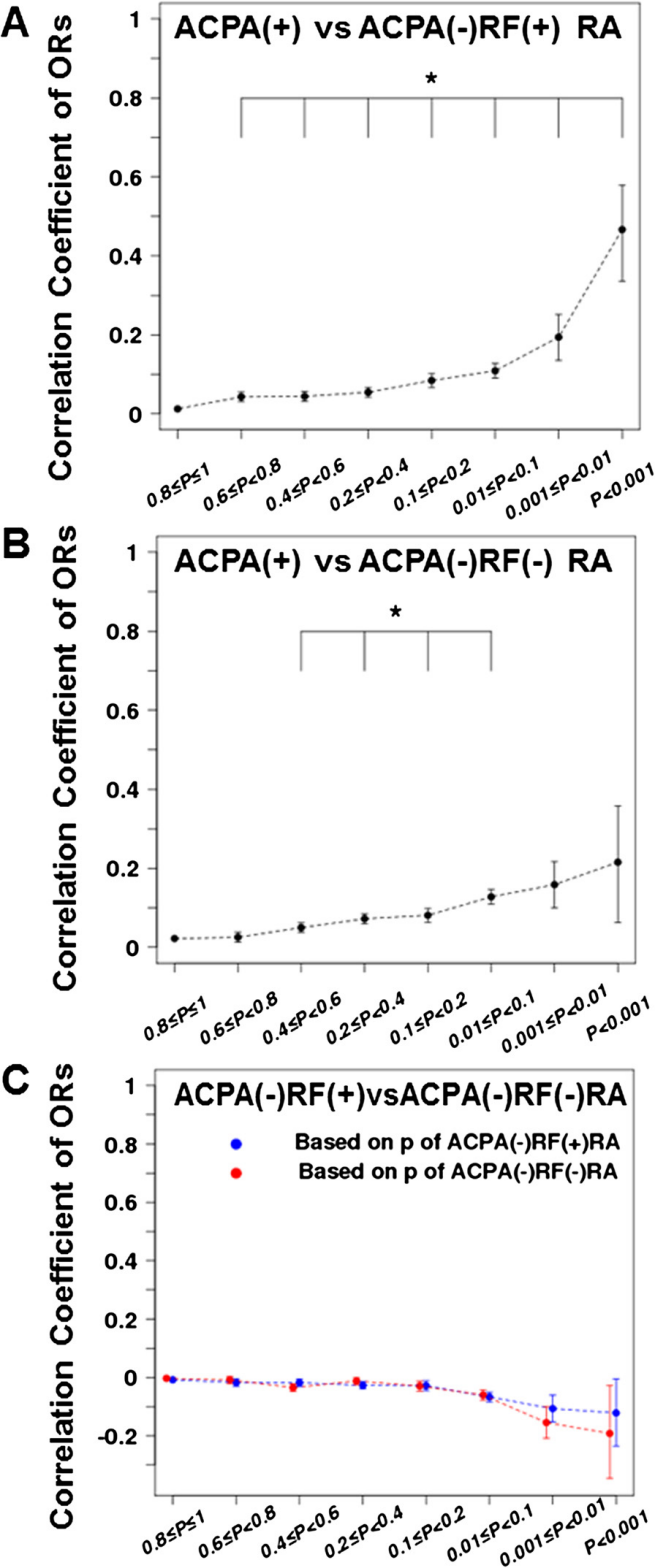
Correlations of odds ratios (ORs) in sets of non-human leukocyte antigen (HLA) single nucleotide polymorphisms (SNPs) among anti-citrullinated peptide/protein antibody (ACPA)-positive rheumatoid arthritis (RA) and the two subsets of ACPA-negative RA. Correlation coefficient of ORs between ACPA-positive RA and ACPA-negative RF-positive RA **(A)**, between ACPA-positive RA and ACPA-negative RF-negative RA **(B)**, and between ACPA-negative RF-positive RA and ACPA-negative RF-negative RA **(C)**, in terms of SNPs stratified by p-values of ACPA-positive RA **(A, B)** or each study **(C)**. *Positive correlation with *P*-values <1.0 × 10^−7^. SNPs in the HLA locus were excluded from this analysis.

Last, because our recent study showed that ACPA-negative RA consists of two genetically different subsets in terms of HLA-DRB1 usage, that is, ACPA-negative RF-positive RA and ACPA-negative RF-negative RA [32,33], associations of non-HLA markers were compared between these two ACPA-negative subsets. As shown in Figure 3A and B, ORs for SNPs in ACPA-positive RA were correlated with those in ACPA-negative RF-positive RA and ACPA-negative RF-negative RA. In particular, ORs for SNPs showing suggestive associations (*P* <0.001) with ACPA-positive RA also had strong correlation with ACPA-negative RF-positive RA (*r* = 0.47, Figure 3A). On the contrary, when correlations of ORs were analyzed between ACPA-negative RF-positive RA and ACPA-negative RF-negative RA, no positive associations were observed, even for the SNPs suggestive associations (*P* <0.001) (Figure 3C). These results suggest that ACPA-positive RA is genetically similar to ACPA-negative RF-positive RA rather than ACPA-negative RF-negative RA.

## Discussion

Because the number of studies addressing susceptibility loci to ACPA-negative RA including candidate gene analyses is quite limited, this is the first report of GWAS addressing ACPA-negative RA in an Asian population and one of the largest in the world [24,25]. Our study revealed that ACPA-negative RA shares a large proportion of susceptibility loci with ACPA-positive RA for non-HLA alleles. Moreover, our results suggest that ACPA-negative RA consists of two genetically distinct subsets based on RF positivity even for non-HLA genes. Our study participants showed 45.5% of positivity of RF, compatible to the previous ACPA-negative RA cohorts [9].

We confirmed that inclusion of age and sex as covariates did not alter the results. This means that as the prevalence of RA is around 1% and the controls are not young, the influence of contamination of subjects among controls who develop RA in the future should be very small.

Our study confirmed an association between ACPA-negative RA and the HLA locus and revealed suggestive associations of *FCRL3* on chromosome 1 and *CSMD1* on chromosome 8. We did not find heterogeneity among the studies, indicating that these associations were not obtained by one or two studies with extreme association. While the female ratio was different between cases and controls, we did not adjust for sex. This is because most of the previous GWAS, including our previous meta-analysis, did not adjust for sex to analyze autosomal SNPs.

Because rs6904716 in *the HLA locus* was not very strongly associated with ACPA-positive RA in the RA meta-analysis [14], these results suggest that associated HLA alleles are different between ACPA-positive and ACPA-negative RA. Low effect sizes of the HLA locus to ACPA-negative RA may explain the lack of significant association of the HLA locus in the meta-analysis of GWAS.

*FCRL3* has been reported to be associated with RA, especially in the Japanese population [38]. Rs7528684 is considered to be the causative variant of the *FCRL3* region to which NFκB binds and activates B cells to produce antibody by augmenting BCR-mediating signals [38]. Rs17727339 is in moderate linkage disequilibrium (LD) with rs7528684 (D’:0.80 and *r*^2^:0.28). Although the association of rs17727339 in the combined study did not reach the GWAS significant level, the replication study supported the association of *FCRL3* with ACPA-negative RA. Thus, it is likely that the *FCRL3* region, possibly as a consequence of association of rs7528684, is associated with both ACPA-positive and -negative RA.

*CSMD1* is a tumor-suppressor gene associated with psoriasis, Kawasaki disease and schizophrenia [39-41]. *CSMD1* expresses mainly in epithelial cells and exhibits anti-tumor activity through activation of the Smad pathway [42,43]. *CSMD1* also functions as a complement regulatory gene, especially of the classical pathway [43]. Thus, the role of *CSMD1* on complement or other genes nearby *CSMD1* may have an important role on the development of ACPA-negative RA. This chromosome 8 region was not associated with ACPA-positive RA in the RA meta-analysis (*P* = 0.87), therefore, this region may be an ACPA-negative RA-specific associated region. Considering the moderate heterogeneity of rs6986423, further replication studies would confirm the association between this region and ACPA-negative RA.

Four regions out of the thirteen genes that had been shown to be associated with ACPA-negative RA in European populations had *P*-values <0.01 (Additional file 3) in the Japanese population. Although these markers did not reach the stringent significance level, the current results suggest that ACPA-negative RA shares susceptibility loci beyond ethnicity.

Although we did not find ACPA-negative RA-associated genes with a GWAS-significance level due to the limited power of case subjects, the current results suggested that the majority of non-HLA susceptibility loci are shared between ACPA-positive and ACPA-negative RA. Correlation analysis of the 21 SNPs in the susceptibility loci to Japanese RA showed significant correlations of ORs between ACPA-positive and ACPA-negative RA in both the GWAS meta-analysis and the replication study. These consistent correlations support similarity of genetic background between the two RA subsets. Such results are consistent with the previous US report that compared the ORs of representative SNPs in 29 RA susceptibility loci of European ancestry between ACPA-positive RA and ACPA-negative RA. We also showed that the ORs for SNPs weakly associated with ACPA-positive RA are correlated with ACPA-negative RA, with the strength of correlation depending on the strength of the *P*-values for the association. While the UK study emphasized the categories of susceptibility loci (for example, some genes are associated only with ACPA-positive RA and some genes with both ACPA-positive and ACPA-negative RA), we assume that the majority of the susceptibility loci are shared between ACPA-positive and ACPA-negative RA. Because the results of susceptibility analysis for genes with a small effect size vary by the sample size, correlation analysis of effect size may be more powerful than the orthodox association analysis in such cases. In fact, when we calculated the correlation of ORs for the 35 non-HLA SNPs from the table in the UK study [27], the correlation coefficient was 0.63, showing that many of the genes are shared between ACPA-positive and -negative RA. These strong correlations between ACPA-positive and -negative RA matches with the current study as well as the US study [31].

On the contrary, HLA-association seems to be different between ACPA-positive and -negative RA. We and others have already shown that the HLA-DRB1 allele usage in ACPA-negative RA is different from that in ACPA-positive RA [5-9]. The current study confirmed the previous results, including those of the UK studies [23,25], in that rs6904716, which had the smallest *P*-value for ACPA-negative RA in the current study, did not show strong association in ACPA-positive RA in the RA meta-analysis (*P* = 2.3 × 10^−15^) compared with other SNPs in the HLA locus (the smallest *P* = 1.2 × 10^−130^), whereas rs7764819, displaying the strongest association in the HLA locus with ACPA-positive RA in the RA meta-analysis, did not even show a suggestive association with ACPA-negative RA in the current study (*P* = 0.56). All these results confirmed the idea that ACPA-negative RA uses the different HLA allele from ACPA-positive RA. These results may suggest that T cells in ACPA-positive RA react against relatively uniform autoantigens, citrullinated proteins, whereas T cells in ACPA-negative RA react with varied autoanitigens.

In the current study, we also confirmed that the two subsets of ACPA-negative RA, ACPA-negative RF-positive RA and ACPA-negative RF-negative RA, are genetically distinct. Previously we reported that the HLA allele usage is different between the two ACPA-negative RA subsets [30]. Here we showed that not only HLA allele usage but also the association of non-HLA genes is different (Figure 3C). As Figure 3A and B show, ACPA-negative RF-positive RA is genetically closer to ACPA-positive RA than ACPA-negative RF-negative RA. Therefore, only ACPA-negative RF-negative RA may be a relatively different subset from conventional RA including ACPA-negative RF-positive RA (see Additional file 8).

Because ACPA-negative RA represents a minor subset of RA, it is difficult to perform the association analysis to detect common variants with small effect sizes. In such cases, correlation analysis of effect size of each SNP may be more powerful to determine whether two different subsets share the majority of susceptibility loci or not. We assume that many of the susceptibility loci are shared except for the HLA allele between ACPA-positive and -negative RA, but worldwide meta-analysis would be necessary to confirm this idea.

## Conclusions

Two non-HLA loci showed suggestive associations with ACPA-negative RA in the Japanese population. ACPA-negative RA, especially the RF-positive subset, shares a common genetic background with ACPA-positive RA.

## Additional files

Additional file 1:
**Study design of the current study.** A flow of the current study design is indicated. Additional file 2:
**QQ plot of the genome-wide association studies (GWAS) meta-analysis.** The observed and expected *P*-values are indicated. Additional file 3:
**Association of 13 regions reported to be associated with anti-citrullinated peptide/protein antibody (ACPA)-negative rheumatoid arthritis (RA) in European populations.** The results of the best *P*-values in the 13 regions reported to be associated with ACPA-negative RA in European populations are indicated. Additional file 4:
**Strong correlations of effect sizes in single nucleotide polymorphisms (SNPs) between analyses with or without age and sex as covariates.** The effect sizes in SNPs pruned by linkage disequilibrium (*r* ^2^ > 0.3) are compared between analysis with or without age and sex as covariates and plotted. Additional file 5:
**Manhattan plot of the genome-wide association studies (GWAS) meta-analysis using age and sex as covariates.** The results of the meta-analysis are plotted according to the chromosomal positions and *P*-values. Additional file 6:
**Replication results for all of the regions showing** ***P*** **-values <0.0001 in the genome-wide association studies (GWAS) meta-analysis.** The combined results are also indicated. Additional file 7:
**Results of the 21 single nucleotide polymorphisms (SNPs) in the regions associated with RA in anti-citrullinated peptide/protein antibody (ACPA)-positive and -negative RA.** The results in ACPA-positive and -negative rheumatoid arthritis (RA) are indicated for the 21 SNPs in the regions associated with RA in the RA genome-wide association studies (GWAS) meta-analysis. Additional file 8:
**Schematic image of overlapping of susceptibility alleles among anti-citrullinated peptide/protein antibody (ACPA)-positive and the two subsets of ACPA-negative RA.** Schematic image of overlapping of susceptibility alleles among ACPA-positive and the two subsets of ACPA-negative rheumatoid arthritis (RA) is indicated based on the previous and current results.

## Abbreviations

ACPA: anti-citrullinated peptide/protein antibody
ACR: American College of Rheumatology
ELISA: enzyme-linked immunosorbent assay
GWAS: genome-wide association study
HLA: human leukocyte antigen
LD: linkage disequilibrium
OR: odds ratio
RA: rheumatoid arthritis
RF: rheumatoid factor
SNP: single nucleotide polymorphism
