# Supplementary figures and images for: Anti-citrullinated peptide/protein antibody (ACPA)-negative RA shares a large proportion of susceptibility loci with ACPA-positive RA: a meta-analysis of genome-wide association study in a Japanese population
Source: Arthritis Res Ther. 2015 Apr 18;17(1):104. doi: 10.1186/s13075-015-0623-4 (PMC4431175; doi:10.1186/s13075-015-0623-4)

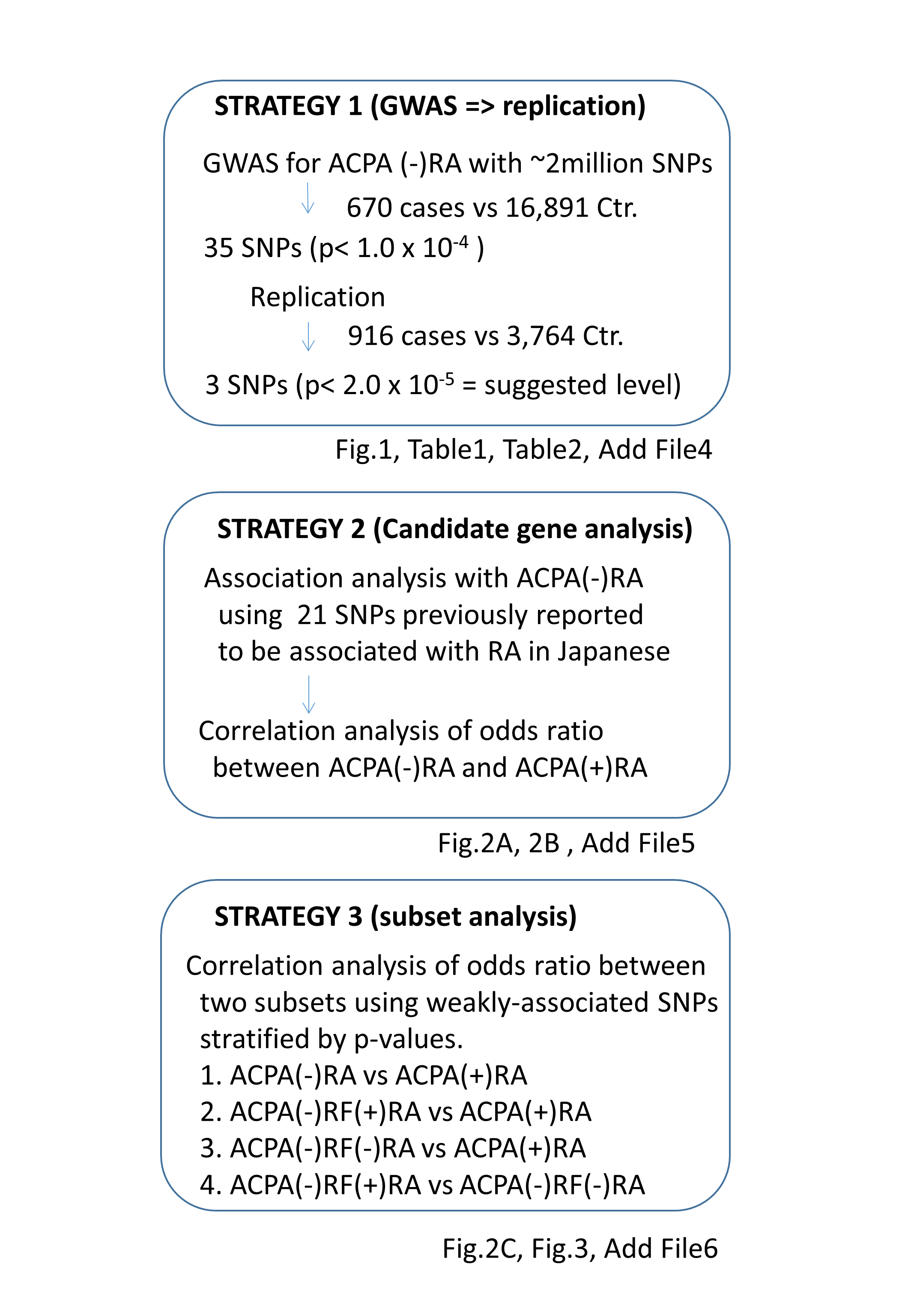

Supplement: Additional file 1: — Study design of the current study. A flow of the current study design is indicated. [file 13075_2015_623_MOESM1_ESM.doc]

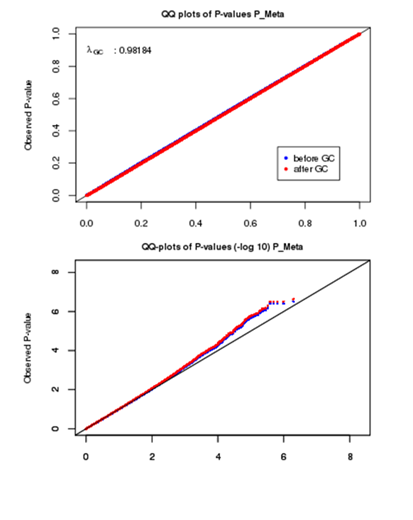

Supplement: Additional file 2: — QQ plot of the genome-wide association studies (GWAS) meta-analysis. The observed and expected P-values are indicated. [file 13075_2015_623_MOESM2_ESM.doc]

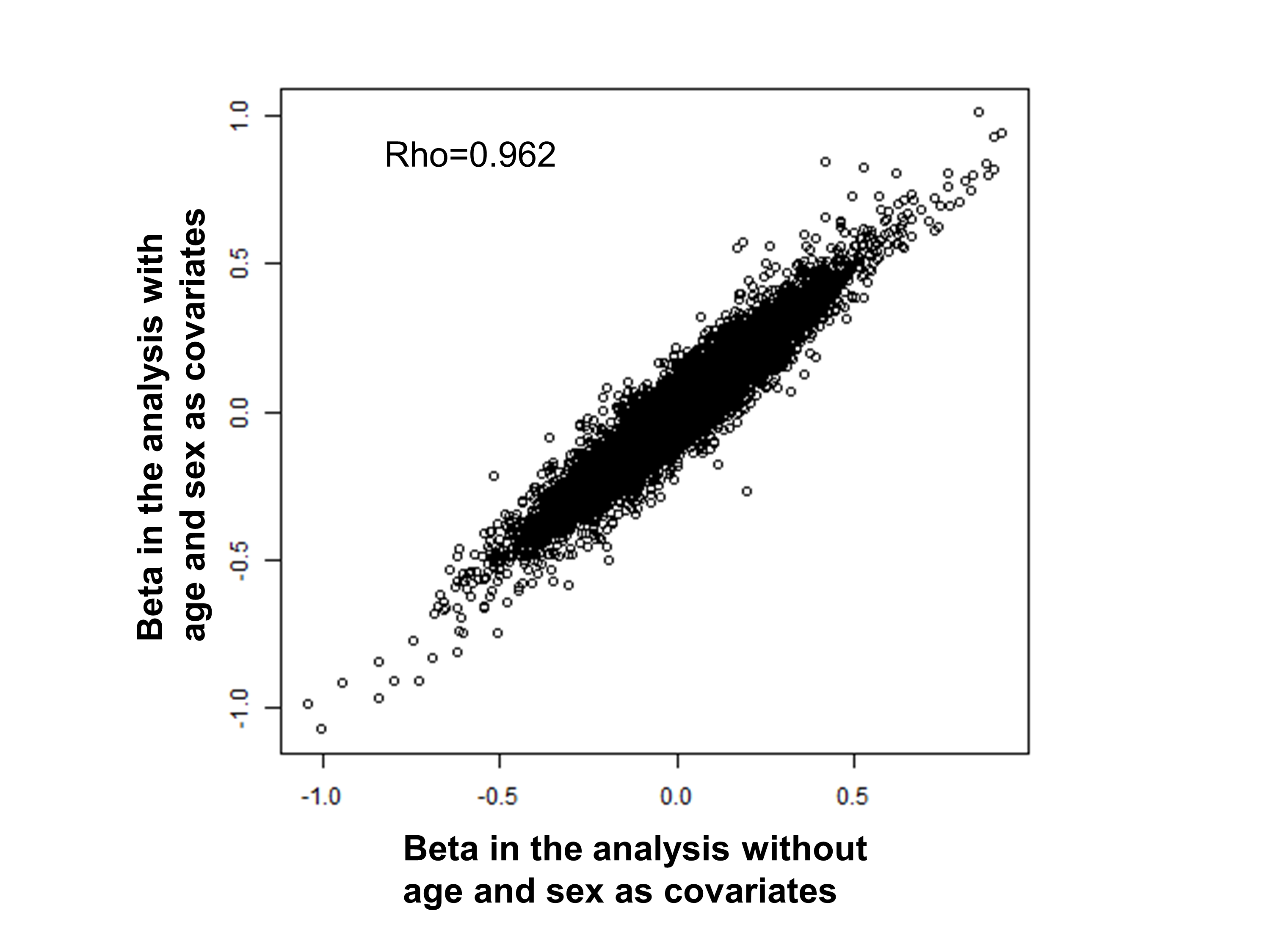

Supplement: Additional file 4: — Strong correlations of effect sizes in single nucleotide polymorphisms (SNPs) between analyses with or without age and sex as covariates. The effect sizes in SNPs pruned by linkage disequilibrium (r 2 > 0.3) are compared between analysis with or without age and sex as covariates and plotted. [file 13075_2015_623_MOESM4_ESM.doc]

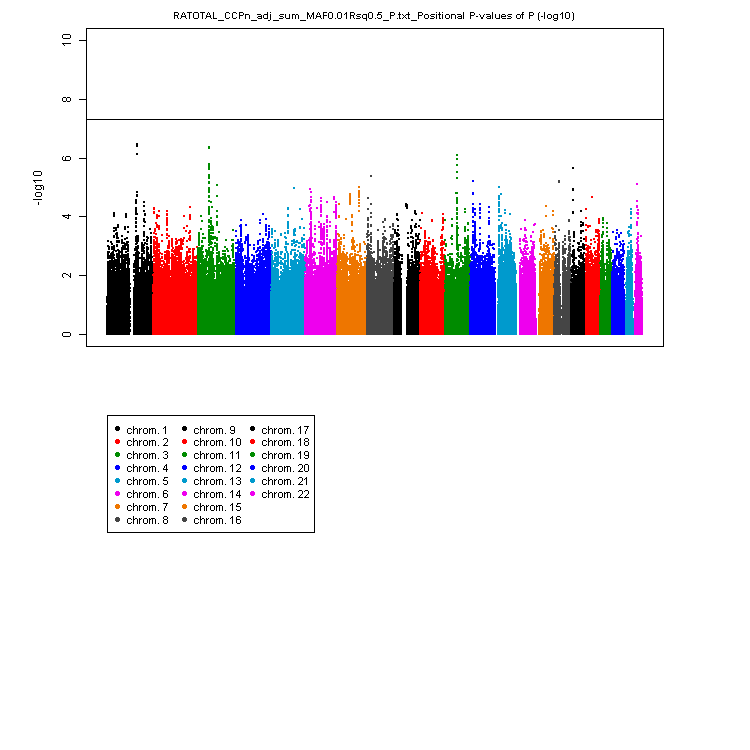

Supplement: Additional file 5: — Manhattan plot of the genome-wide association studies (GWAS) meta-analysis using age and sex as covariates. The results of the meta-analysis are plotted according to the chromosomal positions and P-values. [file 13075_2015_623_MOESM5_ESM.doc]

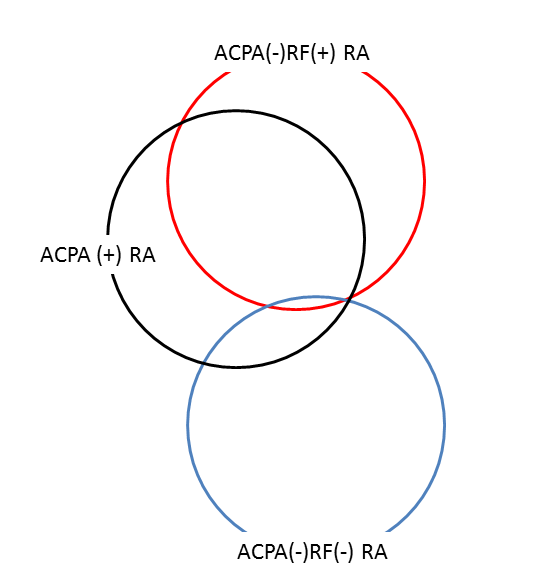

Supplement: Additional file 8: — Schematic image of overlapping of susceptibility alleles among anti-citrullinated peptide/protein antibody (ACPA)-positive and the two subsets of ACPA-negative RA. Schematic image of overlapping of susceptibility alleles among ACPA-positive and the two subsets of ACPA-negative rheumatoid arthritis (RA) is indicated based on the previous and current results. [file 13075_2015_623_MOESM8_ESM.doc]
