# Supplementary material for: Anti-citrullinated peptide/protein antibody (ACPA)-negative RA shares a large proportion of susceptibility loci with ACPA-positive RA: a meta-analysis of genome-wide association study in a Japanese population
Source: Arthritis Res Ther. 2015 Apr 18;17(1):104. doi: 10.1186/s13075-015-0623-4 (PMC4431175; doi:10.1186/s13075-015-0623-4)
Supplement: Additional file 3: — Association of 13 regions reported to be associated with anti-citrullinated peptide/protein antibody (ACPA)-negative rheumatoid arthritis (RA) in European populations. The results of the best P-values in the 13 regions reported to be associated with ACPA-negative RA in European populations are indicated. [file 13075_2015_623_MOESM3_ESM.doc]

| Gene | SNP | Chr | Position | Ref | Var | Beta_Meta | SE_Meta | P_Meta | OR_Meta(95%CI) |
| --- | --- | --- | --- | --- | --- | --- | --- | --- | --- |
| *PTPN22* | rs1746853 | 1 | 114184620 | A | C | 0.197 | 0.057 | 0.00053 | 1.22 (1.09-1.36) |
| *VTCN1* | rs1892408 | 1 | 117491445 | C | G | 0.303 | 0.104 | 0.0035 | 1.35 (1.1-1.67) |
| *STAT4* | rs11694530 | 2 | 191703487 | C | T | 0.161 | 0.080 | 0.044 | 1.17 (1-1.38) |
| *ANKRD55/IL6ST* | rs149140 | 5 | 55552745 | C | T | 0.189 | 0.103 | 0.067 | 1.21 (0.98-1.48) |
| *GIN1/C5orf30* | rs461269 | 5 | 102597523 | A | T | 0.496 | 0.208 | 0.017 | 1.64 (1.08-2.49) |
| *TNFAIP3* | rs661561 | 6 | 138239024 | A | C | -0.239 | 0.082 | 0.0036 | 0.79 (0.67-0.93) |
| *IRF5* | rs3807306 | 7 | 128367916 | G | T | 0.119 | 0.067 | 0.075 | 1.13 (0.99-1.29) |
| *BLK* | rs2618481 | 8 | 11391506 | C | T | 0.112 | 0.063 | 0.076 | 1.12 (0.99-1.27) |
| *CLEC4A* | rs2024301 | 12 | 8169448 | A | T | 0.0360 | 0.059 | 0.54 | 1.04 (0.92-1.17) |
| *CLYBL* | rs9557286 | 13 | 99199677 | A | G | 0.121 | 0.057 | 0.032 | 1.13 (1.01-1.26) |
| *CLEC16A* | rs6498146 | 16 | 11014208 | C | T | -0.246 | 0.082 | 0.0029 | 0.78 (0.66-0.92) |
| *STAT3* | rs17593222 | 17 | 37766516 | C | G | 0.246 | 0.167 | 0.14 | 1.28 (0.91-1.79) |
| *SMIM21* | rs4410169 | 18 | 71267828 | A | G | 0.081 | 0.061 | 0.19 | 1.08 (0.96-1.22) |
