# Supplementary material for: Anti-citrullinated peptide/protein antibody (ACPA)-negative RA shares a large proportion of susceptibility loci with ACPA-positive RA: a meta-analysis of genome-wide association study in a Japanese population
Source: Arthritis Res Ther. 2015 Apr 18;17(1):104. doi: 10.1186/s13075-015-0623-4 (PMC4431175; doi:10.1186/s13075-015-0623-4)
Supplement: Additional file 6: — Replication results for all of the regions showing P -values <0.0001 in the genome-wide association studies (GWAS) meta-analysis. The combined results are also indicated. [file 13075_2015_623_MOESM6_ESM.doc]

| SNP | Chr | Position | Gene | Ref | Var | GWAS Meta-analysis | | | Replication | | | Combined Study | | ACPA pos Meta-analysis | |
| --- | --- | --- | --- | --- | --- | --- | --- | --- | --- | --- | --- | --- | --- | --- | --- |
|  |  |  |  |  |  | Beta | SE | P | Beta | SE | P | P | OR | P | OR |
| rs17727339 | 1 | 155946738 | *FCRL3* | C | T | -0.262 | 0.066 | 8.0 x10^-5^ | -0.142 | 0.063 | 0.025 | 1.4 x10^-5^ | 0.82 (0.75-0.90) | 0.57 | 0.98 (0.91-1.05) |
| rs12410250 | 1 | 197501366 | *LOC647201* | C | T | -0.266 | 0.068 | 9.9 x10^-5^ | 0.023 | 0.064 | 0.72 | 0.016 | 0.89 (0.82-0.98) | 0.31 | 0.96 (0.89-1.04) |
| rs12141283 | 1 | 203187881 | *NFASC* | A | G | 0.390 | 0.093 | 2.7 x10^-5^ | 0.091 | 0.095 | 0.34 | 2.3 x10^-4^ | 1.28 (1.12-1.46) | 0.59 | 0.97 (0.86-1.09) |
| rs16865276 | 2 | 6568763 | *LOC391349* | C | T | 0.292 | 0.075 | 8.9 x10^-5^ | -0.009 | 0.076 | 0.91 | 0.0067 | 1.15 (1.04-1.28) | 0.13 | 1.07 (0.98-1.16) |
| rs10495554 | 2 | 7379418 | *LOC644232* | G | T | -0.227 | 0.057 | 8.0 x10^-5^ | 0.117 | 0.053 | 0.027 | 0.29 | 0.96 (0.89-1.04) | 0.61 | 0.98 (0.93-1.04) |
| rs9036 | 3 | 14505725 | *SLC6A6* | A | G | 0.425 | 0.095 | 7.2 x10^-6^ | 0.102 | 0.09 | 0.257 | 9.2 x10^-5^ | 1.29 (1.13-1.47) | 0.30 | 1.06 (0.95-1.19) |
| rs4974139 | 3 | 56407028 | *CAST1* | C | T | -0.424 | 0.084 | 4.0 x10^-7^ | -0.057 | 0.087 | 0.516 | 4.0 x10^-5^ | 0.78 (0.69-0.88) | 0.28 | 1.06 (0.96-1.17) |
| rs7706034 | 5 | 90396817 | *MASS1* | A | G | 0.236 | 0.057 | 3.9 x10^-5^ | -0.134 | 0.053 | 0.011 | 0.36 | 1.04 (0.96-1.12) | 0.64 | 1.01 (0.96-1.08) |
| rs3129963 | 6 | 32488186 | *BTNL2* | A | G | 0.401 | 0.1 | 5.9 x10^-5^ | 0.105 | 0.102 | 0.303 | 3.4 x10^-4^ | 1.29 (1.12-1.49) | 2.6 x10^-10^ | 0.63 (0.54-0.72) |
| rs3135363 | 6 | 32497626 | *LOC646668* | A | G | 0.241 | 0.06 | 6.8 x10^-5^ | 0.089 | 0.057 | 0.118 | 1.1 x10^-4^ | 1.17 (1.08-1.28) | 7.2 x10^-7^ | 0.84 (0.79-0.90) |
| rs6904716 | 6 | 33849267 | *LEMD2* | A | G | -0.309 | 0.074 | 2.7 x10^-5^ | -0.219 | 0.061 | 0.00036 | 5.7 x10^-8^ | 0.77 (0.70-0.85) | 2.3 x10^-15^ | 0.73 (0.68-0.79) |
| rs944664 | 6 | 85945694 | *LOC643851* | C | T | -0.25 | 0.058 | 1.6 x10^-5^ | 0.02 | 0.054 | 0.702 | 0.0080 | 0.90 (0.83-0.97) | 0.43 | 0.98 (0.92-1.04) |
| rs10499094 | 6 | 120163552 | *LOC644408* | A | G | 0.328 | 0.082 | 6.7 x10^-5^ | -0.032 | 0.087 | 0.715 | 0.0080 | 1.17 (1.04-1.32) | 0.84 | 1.01 (0.92-1.11) |
| rs10484530 | 6 | 167381552 | *FGFR1OP* | C | T | 0.233 | 0.058 | 5.5 x10^-5^ | -0.122 | 0.056 | 0.029 | 0.22 | 1.05 (0.97-1.14) | 7.6 x10^-10^ | 1.21 (1.14-1.29) |
| rs9691397 | 7 | 11944059 | *LOC221981* | C | T | 0.228 | 0.057 | 7.2 x10^-5^ | 0.086 | 0.054 | 0.112 | 1.1 x10^-4^ | 1.16 (1.08-1.26) | 0.34 | 0.97 (0.92-1.03) |
| rs992125 | 7 | 70870928 | *CALN1* | C | T | 0.259 | 0.057 | 6.6 x10^-6^ | -0.022 | 0.053 | 0.683 | 0.0057 | 1.11 (1.03-1.20) | 0.71 | 1.01 (0.95-1.07) |
| rs2254595 | 7 | 120794485 | *FAM3C* | C | T | -0.373 | 0.078 | 1.7 x10^-6^ | -0.004 | 0.078 | 0.961 | 6.5 x10^-4^ | 0.83 (0.74-0.93) | 0.87 | 1.01 (0.92-1.10) |
| rs6986423 | 8 | 4621823 | *CSMD1* | G | T | -0.248 | 0.057 | 1.4 x10^-5^ | -0.128 | 0.053 | 0.017 | 2.4 x10^-6^ | 0.83 (0.77-0.90) | 0.87 | 0.99 (0.94-1.06) |
| rs10096421 | 8 | 10869278 | *XKR6* | G | T | -0.311 | 0.076 | 4.0 x10^-5^ | 0.026 | 0.073 | 0.72 | 0.0097 | 0.87 (0.79-0.97) | 0.056 | 0.92 (0.84-1.00) |
| rs3118766 | 9 | 96065119 | *LOC643240* | G | T | -0.235 | 0.059 | 6.1 x10^-5^ | -0.018 | 0.055 | 0.75 | 0.0029 | 0.89 (0.82-0.96) | 0.046 | 0.94 (0.88-1.00) |
| rs11820322 | 11 | 64169467 | *NRXN2* | C | T | 0.303 | 0.062 | 9.0 x10^-7^ | 0.004 | 0.06 | 0.946 | 4.8 x10^-4^ | 1.16 (1.07-1.27) | 0.025 | 1.08 (1.01-1.16) |
| rs2044714 | 11 | 104731481 | *OR2AL1P* | C | T | 0.439 | 0.106 | 3.5 x10^-5^ | 0.056 | 0.081 | 0.489 | 0.0022 | 1.22 (1.07-1.39) | 0.29 | 1.05 (0.96-1.16) |
| rs1612841 | 12 | 12136155 | *BCL2L14* | C | T | -0.235 | 0.057 | 4.0 x10^-5^ | -0.057 | 0.053 | 0.282 | 3.3 x10^-4^ | 0.87 (0.80-0.94) | 0.29 | 1.03 (0.97-1.10) |
| rs11048888 | 12 | 27185042 | *LOC440091* | C | T | -0.319 | 0.081 | 8.9 x10^-5^ | -0.056 | 0.081 | 0.495 | 0.0011 | 0.83 (0.74-0.93) | 0.074 | 1.09 (0.99-1.20) |
| rs7488334 | 12 | 51432999 | *LOC643898* | G | T | 0.269 | 0.063 | 2.2 x10^-5^ | 0.061 | 0.063 | 0.336 | 2.4 x10^-4^ | 1.18 (1.08-1.29) | 0.36 | 0.97 (0.90-1.04) |
| rs12308078 | 12 | 100512146 | *MYBPC1* | A | G | 0.266 | 0.067 | 7.6 x10^-5^ | 0.022 | 0.063 | 0.722 | 0.0030 | 1.15 (1.05-1.26) | 0.44 | 1.03 (0.96-1.11) |
| rs9509261 | 13 | 19986717 | *CRYL1* | C | T | -0.282 | 0.067 | 2.4 x10^-5^ | 0.027 | 0.056 | 0.631 | 0.020 | 0.91 (0.83-0.99) | 0.93 | 1 (0.94-1.07) |
| rs11073390 | 15 | 93136860 | *LOC440311* | C | T | 0.228 | 0.058 | 7.9 x10^-5^ | 0.012 | 0.055 | 0.822 | 0.0040 | 1.12 (1.04-1.21) | 0.38 | 1.03 (0.97-1.09) |
| rs874562 | 16 | 25010021 | *LOC646939* | C | T | 0.288 | 0.07 | 3.9 x10^-5^ | -0.037 | 0.058 | 0.532 | 0.031 | 1.10 (1.01-1.21) | 0.75 | 0.99 (0.93-1.06) |
| rs7192795 | 16 | 27078719 | *LOC124199* | C | T | -0.418 | 0.104 | 6.2 x10^-5^ | -0.094 | 0.1 | 0.344 | 5.6 x10^-4^ | 0.78 (0.68-0.90) | 0.035 | 0.87 (0.77-0.99) |
| rs937998 | 16 | 84873354 | *DKFZp434O0320* | C | T | -0.414 | 0.104 | 7.2 x10^-5^ | -0.169 | 0.099 | 0.087 | 7.2 x10^-5^ | 0.75 (0.65-0.87) | 0.20 | 0.92 (0.81-1.04) |
| rs2247382 | 17 | 10801962 | *LOC644139* | A | G | -0.264 | 0.065 | 5.3 x10^-5^ | 0.009 | 0.056 | 0.866 | 0.013 | 0.90 (0.83-0.98) | 0.62 | 1.02 (0.95-1.08) |
| rs3889769 | 18 | 33177019 | *BRUNOL4* | C | T | -0.243 | 0.057 | 2.3 x10^-5^ | NA | NA | NA | NA | NA | 0.76 | 0.99 (0.93-1.05) |
| rs2584475 | 18 | 71453209 | *LOC284274* | C | T | -0.278 | 0.071 | 8.3 x10^-5^ | -0.106 | 0.061 | 0.079 | 9.8 x10^-5^ | 0.84 (0.76-0.92) | 0.85 | 0.99 (0.93-1.06) |
| rs2837284 | 21 | 40197271 | *PCP4* | A | G | 0.373 | 0.094 | 6.8 x10^-5^ | 0.064 | 0.073 | 0.38 | 0.0017 | 1.20 (1.07-1.34) | 0.79 | 1.01 (0.93-1.10) |
| rs4321459 | 22 | 25505038 | *CRYBA4* | C | T | -0.252 | 0.059 | 1.8x10^-5^ | -0.025 | 0.054 | 0.637 | 0.0012 | 0.88 (0.81-0.95) | 0.27 | 1.03 (0.98-1.10) |
