# Supplementary material for: Anti-citrullinated peptide/protein antibody (ACPA)-negative RA shares a large proportion of susceptibility loci with ACPA-positive RA: a meta-analysis of genome-wide association study in a Japanese population
Source: Arthritis Res Ther. 2015 Apr 18;17(1):104. doi: 10.1186/s13075-015-0623-4 (PMC4431175; doi:10.1186/s13075-015-0623-4)
Supplement: Additional file 7: — Results of the 21 single nucleotide polymorphisms (SNPs) in the regions associated with RA in anti-citrullinated peptide/protein antibody (ACPA)-positive and -negative RA. The results in ACPA-positive and -negative rheumatoid arthritis (RA) are indicated for the 21 SNPs in the regions associated with RA in the RA genome-wide association studies (GWAS) meta-analysis. [file 13075_2015_623_MOESM7_ESM.doc]

| SNP | Chr | Position | Gene | Allele | | ACPA(+) GWAS meta-analysis | | | ACPA(-) GWAS Meta-analysis | | | ACPA(-) Replication | | | ACPA(-) Combined Study | |
| --- | --- | --- | --- | --- | --- | --- | --- | --- | --- | --- | --- | --- | --- | --- | --- | --- |
|  |  |  |  | Ref | Var | Beta | SE | P | Beta | SE | P | Beta | SE | P | P | OR |
| rs3890745 | 1 | 2543484 | *MMEL1* | C | T | 0.099 | 0.030 | 0.0010 | 0.082 | 0.057 | 0.15 | -0.082 | 0.053 | 0.12 | 0.88 | 0.99 (0.92-1.07) |
| rs2240335 | 1 | 17547124 | *PADI4* | A | C | 0.141 | 0.030 | 5.3 x10^-6^ | 0.09 | 0.058 | 0.12 | 0.088 | 0.053 | 0.097 | 0.023 | 1.09 (1.01-1.18) |
| rs17727339 | 1 | 155946738 | *FCRL3* | C | T | -0.021 | 0.037 | 0.57 | -0.26 | 0.066 | 8.0 x10^-5^ | -0.142 | 0.063 | 0.025 | 1.4x10^-5^ | 0.82 (0.75-0.90) |
| rs11900673 | 2 | 62306165 | *B3GNT2* | C | T | 0.112 | 0.033 | 7.4 x10^-4^ | 0.201 | 0.061 | 0.0011 | 0.05 | 0.059 | 0.39 | 0.0037 | 1.13 (1.04-1.23) |
| rs1876518 | 2 | 65462413 | *SPRED2* | C | T | 0.178 | 0.038 | 5.5 x10^-6^ | 0.129 | 0.074 | 0.082 | -0.094 | 0.071 | 0.18 | 0.80 | 1.01 (0.91-1.12) |
| rs6740838 | 2 | 100179931 | *AFF3* | G | T | 0.094 | 0.030 | 0.0018 | 0.01 | 0.057 | 0.87 | 0.009 | 0.053 | 0.87 | 0.81 | 1.01 (0.93-1.09) |
| rs7574865 | 2 | 191672878 | *STAT4* | G | T | 0.182 | 0.032 | 3.4 x10^-8^ | 0.001 | 0.063 | 0.98 | 0.132 | 0.054 | 0.015 | 0.062 | 1.08 (0.99-1.17) |
| rs926169 | 2 | 204430997 | *CTLA4* | G | T | 0.089 | 0.031 | 0.0048 | 0.107 | 0.060 | 0.073 | -0.034 | 0.055 | 0.53 | 0.45 | 1.03 (0.95-1.12) |
| rs2867461 | 4 | 79732239 | *ANXA3* | A | G | -0.118 | 0.03 | 1.3 x10^-4^ | -0.16 | 0.058 | 0.0047 | -0.053 | 0.054 | 0.32 | 0.0094 | 0.90 (0.83-0.98) |
| rs657075 | 5 | 131458017 | *CSF2* | A | G | -0.117 | 0.031 | 2.1 x10^-4^ | -0.11 | 0.059 | 0.073 | -0.113 | 0.056 | 0.041 | 0.0060 | 0.89 (0.82-0.97) |
| rs12529514 | 6 | 14204637 | *CD83* | C | T | -0.182 | 0.041 | 1.6 x10^-5^ | -0.12 | 0.079 | 0.14 | -0.059 | 0.073 | 0.42 | 0.10 | 0.92 (0.82-1.02) |
| rs2233434 | 6 | 44340898 | *NFKBIE* | A | G | 0.251 | 0.035 | 2.6 x10^-12^ | -0.07 | 0.071 | 0.36 | -0.063 | 0.066 | 0.34 | 0.17 | 0.94 (0.85-1.03) |
| rs2230926 | 6 | 138237759 | *TNFAIP3* | G | T | -0.307 | 0.056 | 6.1 x10^-8^ | -0.16 | 0.110 | 0.15 | -0.238 | 0.098 | 0.015 | 0.0054 | 0.82 (0.70-0.94) |
| rs3093024 | 6 | 167452783 | *CCR6* | A | G | -0.251 | 0.031 | 1.1 x10^-15^ | -0.19 | 0.058 | 0.0012 | -0.086 | 0.053 | 0.10 | 0.00066 | 0.88 (0.81-0.95) |
| rs4731549 | 7 | 128529149 | *IRF5/*  *TNPO3* | C | T | 0.201 | 0.044 | 9.0 x10^-6^ | 0.211 | 0.086 | 0.014 | -0.004 | 0.074 | 0.96 | 0.12 | 1.09 (0.98-1.22) |
| rs2736340 | 8 | 11381382 | *BLK* | C | T | 0.118 | 0.033 | 4.9 x10^-4^ | 0.103 | 0.063 | 0.10 | 0.147 | 0.06 | 0.014 | 0.0037 | 1.13 (1.04-1.24) |
| rs10821944 | 10 | 63455095 | *ARID5B* | G | T | -0.169 | 0.031 | 6.9x10^-8^ | -0.11 | 0.059 | 0.074 | -0.175 | 0.055 | 0.0014 | 0.040 | 0.87 (0.80-0.94) |
| rs3781913 | 11 | 72051144 | *PDE2A/*  *ARAP1* | G | T | 0.119 | 0.033 | 3.7 x10^-4^ | 0.107 | 0.063 | 0.091 | 0.05 | 0.058 | 0.39 | 0.074 | 1.08 (0.99-1.18) |
| rs2841277 | 14 | 104462050 | *PLD4* | C | T | 0.110 | 0.033 | 0.0011 | 0.092 | 0.063 | 0.15 | 0.143 | 0.058 | 0.014 | 0.0051 | 1.13 (1.03-1.23) |
| rs2847297 | 18 | 12787694 | *PTPN2* | A | G | 0.146 | 0.031 | 3.1 x10^-6^ | 0.12 | 0.059 | 0.043 | 0.011 | 0.056 | 0.84 | 0.12 | 1.06 (0.98-1.15) |
| rs2075876 | 21 | 44533581 | *AIRE* | A | G | -0.142 | 0.031 | 8.1 x10^-6^ | -0.08 | 0.060 | 0.20 | 0.053 | 0.056 | 0.35 | 0.83 | 0.99 (0.91-1.08) |
